# Optimization of Mix Proportion and Performance Study of Metakaolin-Slag Geopolymer Mortar Based on Orthogonal Experiment

**DOI:** 10.3390/ma19102004

**Published:** 2026-05-12

**Authors:** Pengchang Liang, Lianyong Zhu, Ruize Yin, Renfei Gao

**Affiliations:** College of Water Hydraulic and Architectural Engineering, Tarim University, Alar 843300, China; 10757232189@stumail.taru.edu.cn (P.L.); 10757232192@stumail.taru.edu.cn (R.Y.); 10757232194@stumail.taru.edu.cn (R.G.)

**Keywords:** metakaolin, slag, orthogonal experiment, mix proportion optimization, repair mortar, microstructural analysis

## Abstract

To promote the practical application of metakaolin-slag geopolymer materials in engineering repair, it is essential to clarify the influence of mix proportion parameters on macroscopic properties, given their inherent deficiencies of inferior toughness and volume stability. In this study, a five-factor and four-level orthogonal experimental design was adopted to systematically investigate the effects of slag content, water glass modulus, alkali equivalent, water–binder ratio, and sand–binder ratio on the fluidity, compressive strength, flexural strength, compressive-to-flexural strength ratio (toughness indicator), and drying shrinkage rate (volume stability indicator) of geopolymer mortar. Range analysis and variance analysis were conducted to clarify the primary and secondary order of influencing factors for each performance index, and the optimal mix proportion balancing multiple performance demands was determined. The results indicate that alkali equivalent is the core factor governing compressive and flexural strength, whereas slag content dominates the compressive-to-flexural ratio, fluidity and drying shrinkage. The geopolymer mortar achieves relatively optimal comprehensive performance when the slag content is 20%, the sodium silicate modulus is 1.6, the alkali equivalent is 12%, the water-to-binder ratio is 0.49, and the sand-to-binder ratio is 2:1, and all indicators meet the specification requirements for rigid repair mortar. Combined with SEM-EDS and XRD microstructural analysis, the main products of the metakaolin-slag system are amorphous N-A-S-H gel and C-(A)-S-H gel. Appropriate alkali equivalent and slag content can promote the dissolution of aluminosilicate raw materials and facilitate the formation of both gel products, providing microstructural support for the improvement of macroscopic performance.

## 1. Introduction

Ordinary Portland cement (OPC) is the most widely used man-made material in the construction field. With its stable mechanical properties and mature preparation technology, it has long dominated the market of civil engineering materials. However, the cement production process consumes large amounts of non-renewable mineral resources such as limestone and emits about 0.8–1.0 ton of CO_2_ per ton of Portland cement clinker produced, accounting for 5–8% of global total CO_2_ emissions [1]. With the comprehensive implementation of the Paris Agreement and the continuous increase in ecological and environmental protection requirements, the resource and environmental constraints of traditional Portland cement are becoming increasingly prominent. The development of green cementitious materials, such as low-energy, low-emission, and resource-reutilizing alternatives, has become a research hotspot and urgent need in the field of construction materials. Some scholars have developed a series of eco-friendly mortars, such as cement-based mortars with waste paper sludge-derived cellulose fibers that improve crack resistance and deformability without sacrificing strength [2].

A geopolymer is a new type of green cementitious material. Its raw materials are mostly metakaolin, fly ash, slag, and other solid wastes. Under the action of an alkaline activator, the raw materials undergo depolymerization-polycondensation reactions to form an inorganic gel with a three-dimensional network structure [3,4,5]. Compared with traditional Portland cement, the carbon emission of geopolymer material production is only 1/5 to 1/3 that of cement, and it enables the resource utilization of industrial solid waste [6]. In addition, geopolymer materials have outstanding advantages such as high early strength, strong corrosion resistance, and excellent high-temperature resistance, showing broad application prospects in building structures, road engineering, repair and reinforcement [7,8,9]. The expansion of their engineering applications has imperative practical significance and strategic value for promoting the green transformation of the construction materials industry and declining carbon emissions.

As the chemical driving force of the geopolymer reaction, the composition and proportion of the alkaline activator directly affect the depolymerization efficiency of aluminosilicate precursors and the structural characteristics of hydration products. Among the various alkaline activators, sodium hydroxide (NaOH) and sodium silicate (Na_2_SiO_3_) are the two most widely used. Although a single NaOH activator can provide a high pH environment, rapidly destroy the structure of silicoaluminate tetrahedra (SiO_4_^4−^, AlO_4_^5−^), and promote precursor depolymerization, the system lacks soluble siloxane groups (SiO_4_^4−^), which can easily delay the polycondensation reaction and result in a loose structure of hydration products. In contrast, sodium silicate can provide a substantial number of soluble siloxane precursors, accelerate the rearrangement and crosslinking of depolymerized products, and promote the formation of a dense 3D gel network, thereby significantly improving the mechanical strength of the geopolymer [10]. Existing studies have confirmed that the composite activation system composed of NaOH and sodium silicate produces a more favorable activating effect than a single activator [11,12]. By optimizing the ratio of the two (i.e., the modulus of sodium silicate) and the total alkali content (alkali equivalent), the synergistic regulation of depolymerization-polycondensation reactions can be achieved, balancing the reaction rate and the structural stability of the products. Hai et al. 2025 [13] found that an appropriate sodium silicate modulus can make the geopolymer microstructure relatively dense, thereby enhancing mechanical strength. Li et al., 2022 [14] pointed out that a suitable alkali equivalent can promote the geopolymer reaction, resulting in more gel products and relatively lower porosity, leading to a denser microstructure and thus greatly improving mechanical properties and water resistance. From the perspective of the reaction mechanism, the introduction of sodium silicate not only supplements the system with active silicon sources, but more importantly, the soluble silicate ions (SiO_4_^4−^) provided can act as structural units in the polycondensation reaction, directly participating in and guiding the construction of the three-dimensional gel network, which is conducive to forming a continuous and dense gel phase. Therefore, this study employs a composite alkaline activator consisting of sodium silicate and NaOH to fully exploit their synergistic effects in promoting depolymerization–polycondensation reactions and optimizing the gel network structure.

The essence of the geopolymer reaction is the depolymerization-polycondensation process of aluminosilicate raw materials in an alkaline environment, and the raw material characteristics directly determine the macroscopic performance [15]. Metakaolin, a classic raw material for geopolymers, is formed by calcining kaolin at 500–800 °C for dehydration. It has highly reactive aluminosilicate components and stable chemical properties, and the resulting geopolymer material has a dense structure and good volume stability [16,17]. However, some studies have shown that metakaolin geopolymer has a slow curing rate under normal temperature curing conditions and insufficient early strength development, limiting its application in the rapid construction scenarios [18,19]. Slag, as a solid waste from the steel industry, is rich in reactive Ca, Si, and Al components in its glassy structure, and its alkali-activated activity is significantly higher than that of metakaolin. Slag-based geopolymers have fast setting and hardening characteristics, and high early strength [20], but single-slag geopolymer has defects such as large drying shrinkage and poor volume stability, which easily lead to early microcracks and affect the long-term durability of the material [21,22,23,24]. Preparing a binary geopolymer by compounding metakaolin and slag can achieve both high early strength and good volume stability through their synergistic effect. The high activity of slag can accelerate the alkali activation reaction process and promote early strength development. Metakaolin can improve the compactness and volume stability of the system and inhibit shrinkage deformation. The combination of the two provides an effective way to regulate the performance of geopolymer materials [25,26]. Metakaolin-slag geopolymer mortar (MSM) exhibits high early strength and excellent bonding performance, thereby demonstrating promising application potential in the repair and strengthening of concrete structures [9]. Nevertheless, existing studies have confirmed that such materials suffer from insufficient flexibility and prominent brittleness, which are manifested as a high compressive-to-flexural strength ratio and high cracking susceptibility. These drawbacks impair the integrity and durability of repaired structural components [27].

In summary, alkali-activated geopolymer mortar fabricated using slag and metakaolin as precursors integrates low-carbon performance and outstanding mechanical strength, which makes it a promising sustainable construction material to substitute traditional cement-based mortar. However, the inherent poor toughness of this material restricts its large-scale application. At present, most studies on this binary geopolymer system focus on the effects of single factors on fluidity, mechanical properties and microstructure of hardened paste, whereas research on the synergistic effects of multiple parameters is relatively insufficient. Meanwhile, few investigations have been conducted on the coordinated improvement of workability, strength, toughness and volume stability through mixture proportion optimization under multi-factor conditions. Against this research background, a five-factor and four-level orthogonal experimental design was adopted in this study to explore the influence mechanisms of slag dosage, water glass modulus, alkali equivalent, water–binder ratio and sand–binder ratio on the fluidity, flexural strength, compressive strength, compressive-to-flexural strength ratio and drying shrinkage of mortar. Afterwards, the optimal mixing ratio was determined with the optimal comprehensive performance as the evaluation target, and it was verified that all performance indicators under this ratio complied with the relevant specification standards for rigid repair mortar. Finally, combined with X-ray diffraction (XRD), scanning electron microscopy-energy dispersive spectroscopy (SEM-EDS) and other microscopic characterization techniques, the phase composition and micro-morphology of different samples were analyzed to clarify the underlying microscopic mechanism. The research outcomes are expected to provide theoretical support and technical guidance for the engineering application of repair mortar.

## 2. Test Materials and Procedures

### 2.1. Test Materials

**Figure 1:**
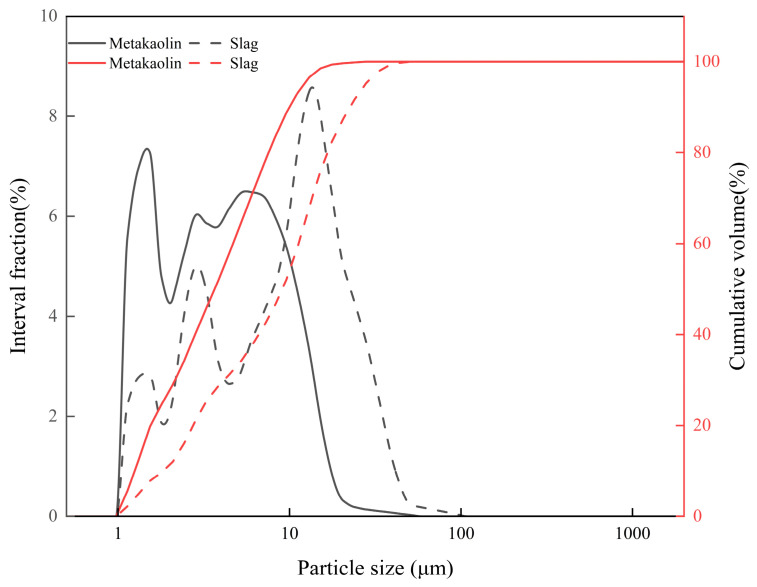
Particle size distributions of the slag and metakaolin.

**Figure 2:**
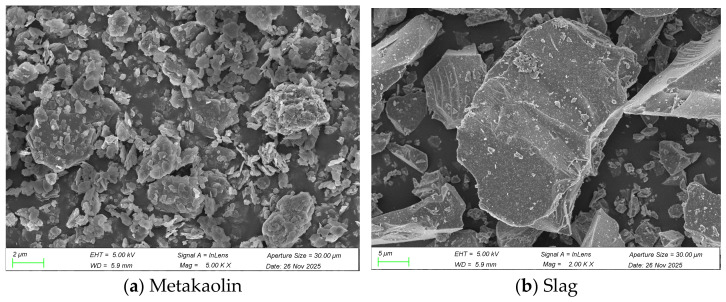
SEM micrographs of the slag and metakaolin.

Metakaolin (MK) and granulated blast furnace slag powder were used as aluminosilicate raw materials in this test. It is an amorphous aluminosilicate material obtained by calcining kaolin at 600–900 °C for dehydroxylation treatment. The metakaolin used in the test was purchased from Chenyu Refractory Abrasive Co., Ltd. in Gongyi City, Henan Province, China, with a sieving fineness of 1250 mesh. It is mainly composed of SiO_2_ and Al_2_O_3_. Granulated blast furnace slag powder (Slag) is a by-product left after blast furnace ironmaking, processed into a fine powder material by granulation, drying, and grinding. According to the activity index, it is mainly divided into three grades: S75, S95, and S105. The slag used in the test was purchased from Henan Borun New Material Co., Ltd., Xinxiang, Henan, China, with a slag grade of S95. Its main chemical components are CaO and SiO_2_. The specific chemical composition of the two materials determined by X-ray fluorescence spectroscopy (XRF) is shown in Table 1. The particle size distributions measured by a Mastersizer 3000 laser particle size analyzer (Malvern Panalytical Ltd., Malvern, Worcestershire, UK) are displayed in Figure 1, in which the D_50_ = 9.093 μm of slag is significantly larger than that of metakaolin (3.652 μm). The scanning electron microscopy at magnifications of 5000× (metakaolin) and 2000× (slag) is shown in Figure 2, using a JSM-IT200 scanning electron microscope (JEOL Ltd., Tokyo, Japan), Metakaolin particles are mainly thin flaky/scaly aggregates with a rough and porous surface, and the particle size is concentrated in the range of 1–5 μm; the slag particles are mainly dense and angular, with sharp edges and a smooth, compact surface. The particle size of slag is larger than that of metakaolin, mainly distributed in the range of 5–20 μm.

The test used sodium silicate (Na_2_SiO_3_) as the alkaline activator, purchased from Yourui Refractory Materials Co., Ltd. in Jiashan County, Zhejiang Province, China. The solution was a transparent viscous liquid with a modulus of 2.3, containing 13.5% sodium oxide and 30% silicon dioxide. Sodium hydroxide was in the form of solid particles with a purity of ≥98%, purchased from Tianjin Zhiyuan Chemical Reagent Co., Ltd., Tianjin Shi, China, and was used to adjust the modulus of sodium silicate to meet the requirements of different test groups for the alkaline activator. The modulus adjustment was calculated using Equations (1) and (2).

(1) $$2\mathrm{NaOH}+\mathrm{Si}O_{2}={\mathrm{Na}}_{2}\mathrm{Si}O_{3}+H_{2}O$$

(2) $$m_{\mathrm{NaOH}}=80\;\times \;m_{0}\;\times \;(\frac{0.3}{60M}-\frac{0.135}{62})$$

In the formula, m_0_ is the mass of the existing sodium silicate (g); M is the target modulus, and m_NaOH_ is the mass of solid NaOH to be added (g).

The fine aggregate used was local natural river sand from Aral city, Xinjiang, China, to be close to practical engineering applications. Its technical indicators were tested in accordance with GB/T 14684-2022 “Sand for Construction” [28]. The bulk density was 2.58 kg/m^3^, and the fineness modulus was 2.54, belonging to medium sand, which is the most commonly used sand in engineering. Table 2 shows the sieve analysis results of the medium sand. The water used in the test was local tap water from Aral City.

### 2.2. Test Design

To guarantee the rationality of the orthogonal experimental design and the generalizability of test results, the selection criteria for each factor and its levels were determined on the basis of preliminary tests and literature references [29,30,31], as described below. The slag dosage (20–80%) covers the proportion range from low incorporation to slag-dominated mixing, to explore the reaction characteristics and performance evolution of the metakaolin-slag composite system under different mixing ratios. The water glass modulus (1.0–1.6) was selected with reference to the commonly adopted activator modulus range for geopolymers (typically 1.0–2.0). Preliminary tests verified that the paste exhibited excessively rapid setting when the modulus was lower than 1.0, thereby setting 1.0 as the lower limit. The alkali equivalent (6–12%) encompasses the conventional dosage range of alkali activators. Pre-experimental results indicated that an alkali equivalent below 6% led to inadequate reaction and low strength, while alkali efflorescence readily occurred when the content exceeded 12%. The water-to-binder ratio (0.43–0.49) was screened and determined to satisfy the molding requirement of initial mortar fluidity without obvious bleeding. The sand-to-binder ratio (1.5–3) corresponds to the typical aggregate–binder proportion for repair mortars, which balances workability and mechanical performance. The above level configurations not only consider the critical influence range of each factor on the properties of geopolymer mortar, but also ensure the representativeness of the orthogonal test and its practical engineering applicability.

The test established a five-factor, four-level L16 (4^5^) orthogonal experiment. Table 3 shows the specific factors and levels set based on preliminary tests and relevant literature. The study involved five influencing factors: slag content (A), sodium silicate modulus (B), alkali equivalent (C), water-to-binder ratio (D), and sand-to-binder ratio (E), with four levels for each factor. Among them, the binder material mass is the sum of the metakaolin and slag masses. The internal blending method is used to calculate the slag content, i.e., the percentage of slag in the total binder mass. The sodium silicate modulus is the molar ratio of SiO_2_ to Na_2_O in the sodium silicate solution. The alkali equivalent is the mass ratio of Na_2_O in sodium silicate to the binder material. The water-to-binder ratio is the ratio of the sum of the water in sodium silicate and the external water added to the total binder mass. The sand-to-binder ratio refers to the mass ratio of sand to binder material.

### 2.3. Specimen Preparation

**Figure 3:**
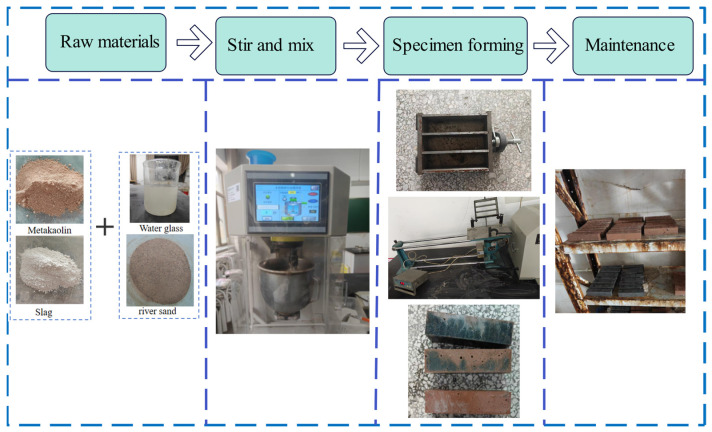
Flow chart of geopolymer mortar specimen preparation.

Figure 3 illustrates the preparation and curing of geopolymer mortar. The alkaline activator was prepared by compounding sodium hydroxide and sodium silicate solution. The required amount of sodium hydroxide particles was dissolved in the sodium silicate solution, and after thorough stirring, it was sealed and stored at room temperature for 24 h before use to ensure the stability and reactivity of the alkaline activator.

The specimens were prepared using a JJ-20H new standard cement mortar mixer (Zhongjiaojianyi Instrument Technology Development Co., Ltd., Beijing, China), and three parallel specimens were set for each mix ratio. The weighed metakaolin and slag powder were placed in the mixing bowl before mixing to ensure uniform distribution of the powders, and the mixer was started at low speed for dry mixing for 30 s to fully blend them. Next, referring to relevant specifications [32], mixing was carried out according to the standard procedure: adding the alkaline activator solution and extra water (low speed for 30 s) → high speed for 30 s (adding sand evenly) → stop mixing for 90 s (scraping) → high speed for 60 s. The mixed mortar was placed into 40 × 40 × 160 mm triple molds pre-coated with release oil in two layers. Each layer was fully tamped to expel air bubbles, and finally, the surface was scraped flat for molding. The specimens were placed in a standard curing room (20 ± 1 °C, relative humidity ≥ 95%) for 24 h, then demolded, numbered, and moved to the curing room to cure until the specified age.

### 2.4. Test Methods

#### 2.4.1. Fluidity Test

The fluidity of the geopolymer mortar was measured in accordance with the Chinese standard GB/T 2419-2005 [33]. Testing was performed using an NLD-3 jumping table (Xinmingsheng Test Instrument Equipment Co., Ltd., Cangzhou City, Hebei Province, China). Each set of experiments is set up with 3 parallel test samples. The geopolymer mortar prepared by the aforementioned method was placed into a truncated cone mold in two layers, with each layer being vertically and evenly tamped 15 times with a tamping rod. After filling, the excess mortar was scraped off and the surface leveled. Next, the mold was vertically lifted, and the flow table was started, making 25 consecutive jumps at a rate of one jump per second. After the jumps stopped, the spreading diameters in two perpendicular directions on the bottom surface of the mortar were immediately measured with a vernier caliper, and the average value was taken as the mortar’s fluidity value.

#### 2.4.2. Mechanical Properties of the Samples

**Figure 4:**
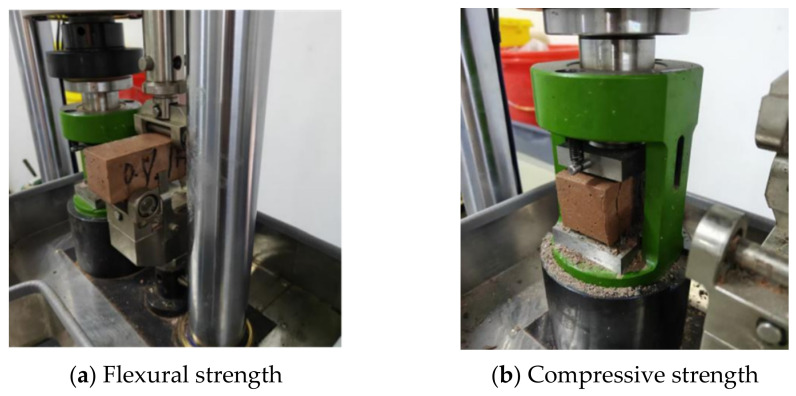
Compressive strength and flexural strength of geopolymer mortar.

The flexural strength and compressive strength of the mortar were measured in accordance with the Chinese standard GB/T 17671-2021 [34] (ISO method). Tests were conducted using an ETF305F-2 electronic compression and flexure testing machine (Wance Testing Machine Co., Ltd., Shenzhen, Guangdong Province, China), as shown in Figure 4a,b. The 40 × 40 × 160 mm prismatic specimens cured under standard conditions to the specified age were taken out. The flexural strength test was conducted first, with the loading rate set at 50 N/s, and the test data at fracture were recorded. Three parallel specimens were taken for each group. After the flexural test, one half of each fractured specimen was placed in a 40 × 40 mm compression fixture and tested for compressive strength on a compression machine at a loading rate of 2.4 kN/s. Six parallel specimens were tested for each group, and the results were expressed as the average value.

**Figure 5:**
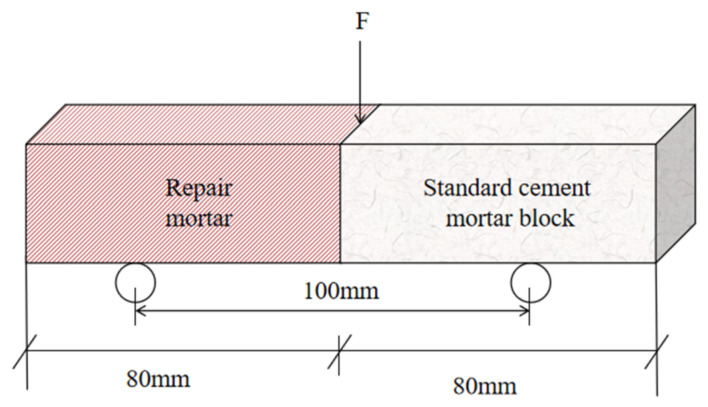
Flexural bond strength test of geopolymer mortar.

The interfacial flexural bond strength test was conducted in accordance with the Chinese standard JC/T 2381-2016 [35]. The substrate specimens were made of PO 42.5 Ordinary Portland cement, with a mix proportion of cement:water:sand = 1:0.5:3 to form 40 × 40 × 160 mm prisms. After 28 days of standard curing, the samples were cut into halves perpendicular to the length direction, and the cut surface was used as the bonding interface. The cut specimen was placed at one end of a triple mold, with the cut surface facing the mold’s center, and the test mortar was cast at the other end, followed by standard curing to the specified age. The bonded specimen was placed on the supports of the flexural testing machine during testing, with the loading point aligned with the interface between the old and new materials, and loaded at a constant rate of 50 N/s until failure, as shown in Figure 5. Six specimens were tested for each group, and the average value was obtained after removing the maximum and minimum values.

#### 2.4.3. Drying Shrinkage Test

The drying shrinkage test was conducted in accordance with the Chinese standard JGJ/T 70-2009 [36], using 40 × 40 × 160 mm prismatic specimens. The specimens cured in the standard curing room for 7 days were removed, the surface was wiped dry, and the initial length was measured using a length comparator. The length comparator used in the test is model SP-176, manufactured by Beijing Zhongjiaojianyi Instrument Technology Development Co., Ltd., Beijing, China. Then the specimens were placed in an environment of 20 ± 2 °C and relative humidity of 60 ± 5% for curing until the specified age, and the length change was measured. The drying shrinkage rate was calculated as the change in length to the initial length value.

#### 2.4.4. Microstructural Analysis Test

The microstructural analysis methods mainly included SEM, EDS, and XRD. For SEM-EDS testing, the specimens cured to the specified age were broken, and small representative pieces were selected. After stopping hydration with anhydrous ethanol, the samples were dried in a vacuum drying oven at 60 °C to a constant weight. The samples were not embedded in resin, but directly fixed with conductive adhesive and subjected to gold sputtering to enhance electrical conductivity. Microscopic morphologies of the samples were observed using a JSM-IT200 scanning electron microscope (JEOL Ltd., Tokyo, Japan). The integrated energy-dispersive X-ray spectrometer (EDS) attached to the instrument was utilized to perform point-scan analysis on typical micro-regions, and relevant EDS data, including the elemental types, atomic percentages, and elemental distribution characteristics of the reaction products, were collected and recorded.

For XRD testing, the specimens cured to the specified age were first broken, and appropriate samples were selected, ground, and passed through a 0.075 mm square-hole sieve. Analysis was performed using an Ultima V X-ray diffractometer (Rigaku Ltd., Tokyo, Japan). The test conditions were as follows: Cu target, scanning range 5–80°, scanning step 0.02°, and scanning rate 2°/min. Qualitative analysis of phases was performed by comparison with standard diffraction patterns (Jade cards).

## 3. Results and Analysis

The test results of the geopolymer mortar according to the orthogonal experimental design scheme are shown in Table 4.

### 3.1. Range Analysis

Based on the orthogonal test results, the average effect and range analysis were performed for each indicator. The average effect value k is the average of the data corresponding to multiple tests of a certain factor at the same level in the orthogonal test, reflecting the test indicator’s variation range. Range analysis is also called direct observation analysis. It refers to the difference R between the maximum and minimum average values among the average values k of all levels of a certain factor. The order of each factor’s primary and secondary influence on each indicator can be determined according to the size of R.

#### 3.1.1. Fluidity

**Figure 6:**
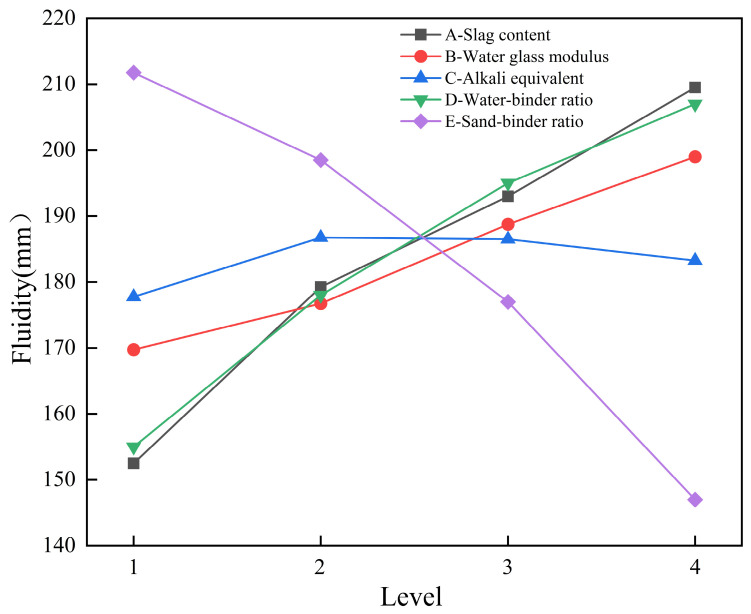
Trend chart of the influence of each factor on mortar fluidity.

Table 5 shows the range analysis results of each factor on the fluidity of geopolymer mortar according to the orthogonal test results. From the size of the range value R, the primary and secondary order of influence of each factor on fluidity is as follows: sand-to-binder ratio (E) > slag content (A) > water-to-binder ratio (D) > sodium silicate modulus (B) > alkali equivalent (C). Figure 6 shows the influence of each factor on mortar fluidity. In addition, the water-to-binder and sand-to-binder ratios exert a pronounced influence on fluidity, which further increases with ameliorating slag content. This is mainly attributed to the difference in the particle morphology of metakaolin and slag. Metakaolin is a flaky/layered clay mineral. Its particles are smaller and have a larger specific surface area than slag, thus the water demand to achieve the same fluidity is higher than that of slag [37,38]. Increasing the sodium silicate modulus relatively reduces the Na_2_O content in the sodium silicate solution, which reduces the solution’s viscosity and increases the paste’s fluidity [39]. Overall, fluidity increases with the increase in slag content, water-to-binder ratio, and sodium silicate modulus, decreases with the increase in sand-to-binder ratio, and alkali equivalent has a relatively small effect on fluidity. The optimal fluidity level is A4B4C2D4E1, i.e., slag content 80%, sodium silicate modulus 1.6, alkali equivalent 8%, water-to-binder ratio 0.49, and sand-to-binder ratio 1.5.

#### 3.1.2. Flexural and Compressive Strength

Table 6 shows the range analysis results of each factor on the mechanical strength of geopolymer mortar according to the orthogonal test results. Comparing the k values of compressive strength and flexural strength at the two ages, the geopolymer mortar exhibits excellent early strength characteristics. The 7 d strength can usually reach 80–90% of the 28 d strength, a proportion significantly higher than the 60–70% of Ordinary Portland cement (PO 42.5 Ordinary Portland cement was adopted. Specimens were prepared at a cement–water–sand mass ratio of 1:0.5:3. The tested 7-day compressive strength and flexural strength were 27.7 MPa and 5.7 MPa, respectively, while the 28-day compressive strength and flexural strength reached 44.8 MPa and 7.8 MPa) [40,41]. This is mainly because its chemical polycondensation reaction replaces the physical hydration reaction of traditional cement. Alkali activation rapidly destroys the structure of raw materials and re-bonds them into a stable 3D inorganic polymer network in a very short time. Coupled with the synergistic hydration of the high-calcium system, it achieves an early strength development rate far exceeding that of ordinary cement under normal temperature curing. The high early strength of geopolymer mortar meets the key performance requirements of repair materials; thus, it has theoretical feasibility in road and structural repair [42].

**Figure 7:**
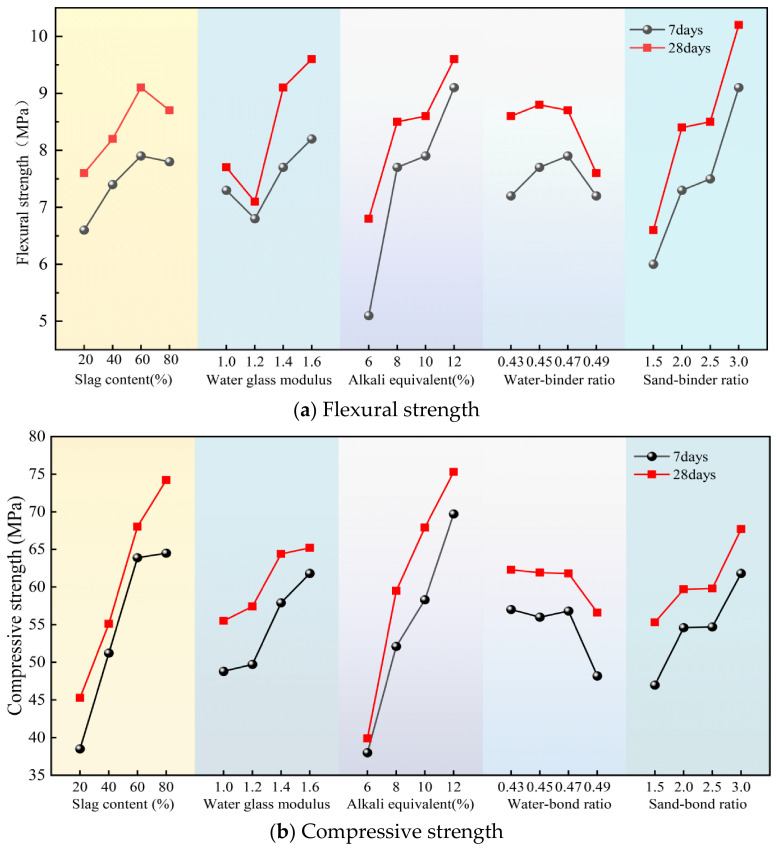
Influence of each factor on mechanical strength.

From the size of the range value R, the primary and secondary order of influence for the 7 d flexural strength of the metakaolin-slag geopolymer mortar is as follows: alkali equivalent (C) > sand-to-binder ratio (E) > sodium silicate modulus (B) > slag content (A) > water-to-binder ratio (D). For 28 d flexural strength, the order of influence factors is E > C > B > A > D. For 7 d and 28 d compressive strengths, the order of influence factors is C > A > E > B > D. Figure 7 shows the trend of the influence of each factor on the mortar’s mechanical strength. The influence of slag content on the flexural and compressive strength of geopolymer mortar shows an obvious difference. As the slag content increases, the mortar’s compressive strength at each age shows a continuous increasing trend, while the flexural strength first increases and then decreases. Both the 7 d and 28 d flexural strengths decrease after the slag content reaches 60%. This can be attributed to the alkali activation activity of slag is better than that of metakaolin. Its incorporation promotes system hydration, leading to increased formation of C–A–S–H gel. Together with the N–A–S–H gel generated from metakaolin hydration, these phases effectively fill pores and enhance matrix densification, resulting in a consistent improvement in compressive strength. However, excessive slag will intensify the early hydration shrinkage of the system, increase internal cracks, and thus cause a decrease in flexural strength [43].

As the sodium silicate modulus increases, the mortar’s compressive strength at each age shows a continuous upward trend overall, and the flexural strength exhibits an initial decrease, which is subsequently followed by an increase. In the low-modulus sodium silicate system, the excessively high OH^−^ concentration gives rise to an overly rapid depolymerization rate of silicoaluminous raw materials at an early stage. This causes the early-stage reaction products to rapidly encapsulate unreacted particles, leading to an insufficient supply of amorphous silicate. When the modulus is increased to 1.6, the amorphous silicate content in the system increases significantly, providing sufficient conditions for the formation of a hydration gel. The alkali equivalent directly determines the system’s pH value. A higher alkali equivalent can provide stronger depolymerization ability for the silicoaluminate tetrahedra, fully activate the activity of metakaolin and slag, greatly increase the amount of hydration gel formed, optimize the microstructure, and thus achieve a steady improvement in mechanical properties [44].

As the water-to-binder ratio increases, both the flexural strength and compressive strength of the mortar first increase slightly and then decline markedly, with an optimal water-to-binder ratio existing. Appropriately increasing the amount of added water can effectively ameliorate the dispersion of the cementitious powder and promote uniform and full hydration reaction in the system. However, excessive additional mixing water evaporates during the hardening process of mortar, leaving a large number of pores inside the matrix. It should be noted that pores are not inherently harmful. However, the pores formed by excessive water addition usually exhibit large pore sizes or interconnected structures, and such pores exert adverse effects on the mechanical strength [45]. As the sand-to-binder ratio increases, both the flexural and compressive strengths of the mortar at each age show a continuous increasing trend. An appropriate amount of quartz sand aggregate can form a rigid skeleton in the hardened matrix, effectively dispersing the stress concentration during loading, inhibiting the development of microcracks, and simultaneously optimizing the interfacial transition zone between the aggregate and the geopolymer matrix [46].

Overall, as the slag content increases, the mortar’s compressive strength continuously increases, while the flexural strength first increases and then decreases, with an optimal content existing. The compressive strength continuously increases as the sodium silicate modulus increases, and the flexural strength first decreases and then increases, with a strength trough in the low-modulus range. As the alkali equivalent and sand-to-binder ratio increase, both the flexural and compressive strength of the mortar at each age increase significantly and continuously. As the water-to-binder ratio increases, both flexural and compressive strength first show a small increase slightly and then decrease significantly, with an optimal water-to-binder ratio existing.

#### 3.1.3. Compressive-to-Flexural Strength Ratio

**Figure 8:**
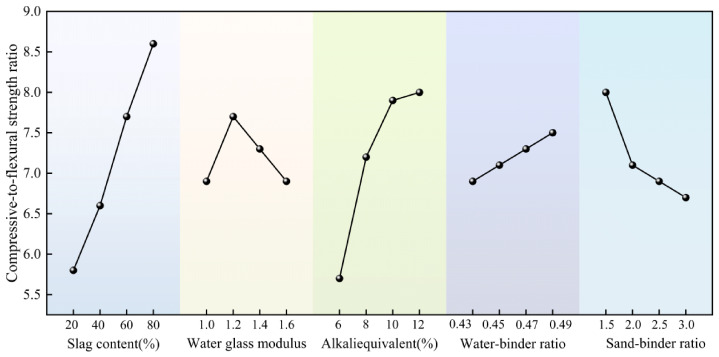
Trend of the influence of each factor on the 28 d compressive-to-flexural strength ratio.

The compressive-to-flexural strength ratio refers to the ratio of compressive strength to flexural strength. As a key indicator of mortar toughness, lower values correspond to reduced brittleness and enhanced toughness [47]. Table 6 shows the range analysis results of each factor on the compressive-to-flexural strength ratio of geopolymer mortar according to the orthogonal test results. From the size of the range value R, the order of influence of each factor on the 28 d compressive-to-flexural strength ratio is as follows: slag content (A) > alkali equivalent (C) > sand-to-binder ratio (E) > sodium silicate modulus (B) > water-to-binder ratio (D). Figure 8 shows the influence of each factor on the mortar’s compressive-to-flexural strength ratio. The compressive-to-flexural strength ratio shows a continuous upward trend with the increase in slag content. This is because the proportion of C-(A)-S-H gel in the system increases as the slag content increases. This gel has stronger brittleness than metakaolin-derived N-A-S-H. Flexural strength is more sensitive to brittleness and stress concentration, and its growth is much slower than that of compressive strength, resulting in increased material brittleness [48]. The alkali equivalent has the most significant effect on the compressive-to-flexural strength ratio, which increases continuously with the increase in the alkali equivalent. Although a high alkali equivalent accelerates the reaction process, it can also increase the brittleness of the gel structure and decrease the uniformity of the interfacial transition zone. This results in flexural strength developing more slowly than compressive strength, leading to an increased compressive-to-flexural strength ratio [9]. Increasing the sand-to-binder ratio increases the compressive-to-flexural strength ratio. As the sand-to-binder ratio increased from 1.5 to 3.0, the compressive-to-flexural strength ratio climbed from 6.7 to 8. The skeletal effect of the aggregate causes compressive strength to increase less than flexural strength, resulting in greater brittleness. The effect of the water-to-binder ratio on the compressive-to-flexural strength ratio is moderate. In the range of 0.45–0.47, the compressive-to-flexural strength ratio is relatively low, and the material toughness is relatively optimal.

The alkali equivalent and slag content are the key factors that control the compressive-to-flexural strength ratio, and an increase in both leading to increase in the brittleness of the material. Excessively high alkali equivalent and excessively high slag content should be avoided to obtain better toughness. The optimal level combination for the compressive-to-flexural strength ratio is A1B4C1D1E4, i.e., slag content 20%, sodium silicate modulus 1.6, alkali equivalent 6%, water-to-binder ratio 0.43, sand-to-binder ratio 3.

#### 3.1.4. Drying Shrinkage Rate

**Figure 9:**
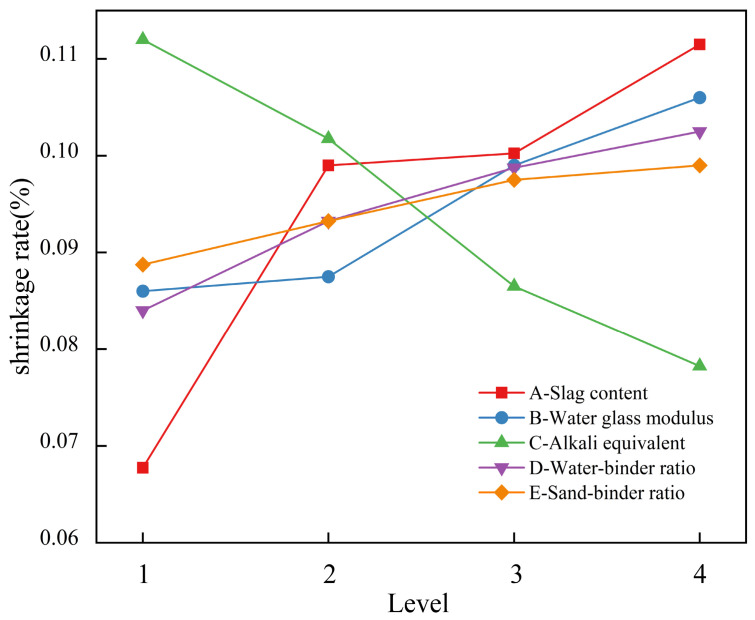
Influence of each factor on the shrinkage rate.

Table 7 shows the range analysis results of each factor on the 28 d drying shrinkage rate of geopolymer mortar according to the orthogonal test results. From the size of the range value R, the order of influence of each factor on the drying shrinkage rate is as follows: slag content (A) > alkali equivalent (C) > sodium silicate modulus (B) > water-to-binder ratio (D) > sand-to-binder ratio (E). Figure 9 shows the influence of each factor on the drying shrinkage rate of the mortar. The mortar’s drying shrinkage rate increases with increasing slag content. This is slag hydration that produces more C-(A)-S-H gel-type products. The C-A-S-H gel has a larger interlayer spacing, and its bound water is easily lost, leading to an enhanced capillary pore shrinkage effect, which further intensifies drying shrinkage deformation, thus increasing the specimen’s overall shrinkage degree [49]. Conversely, as the alkali equivalent increases, the drying shrinkage rate decreases. The main mechanism is that the metakaolin content used in this study has a wide range. Although a high-alkali environment intensifies the reaction and increases chemical shrinkage, it also promotes the reaction of metakaolin, making its dissolution more complete, increasing the amount of N-A-S-H gel generated. Simultaneously, it works synergistically with the C-(A)-S-H gel generated by slag to densify the structure, thereby optimizing the pore structure, refining the pore size distribution, and reducing the proportion of harmful large pores, ultimately resulting in a decrease in drying shrinkage rate [23]. In addition, the drying shrinkage rate of the mortar increases with the water-to-binder ratio. This is attributed to the higher free water content in the system, whose evaporation during drying leaves more pores, enhancing capillary pore shrinkage and intensifying drying shrinkage deformation.

Overall, the drying shrinkage rate of geopolymer mortar increases with increasing slag content, sodium silicate modulus, water-to-binder ratio, and sand-to-binder ratio, whereas it decreases with increasing alkali equivalent. The optimal combination for drying shrinkage rate is A1B1C4D1E1, i.e., slag content 20%, sodium silicate modulus 1.0, alkali equivalent 12%, water-to-binder ratio 0.43, and-to-binder ratio 1.5.

### 3.2. Variance Analysis

To further investigate the influence of each factor on various performance indicators and verify the results of range analysis, variance analysis was performed on each index of the mortar. A five-factor and four-level scheme was adopted for the orthogonal experimental design without blank columns. Accordingly, the sum of squared deviations of the factor with a minor influence was used to substitute the sum of squared errors [50], and the detailed results are listed in Table 8, Table 9, Table 10, Table 11, Table 12, Table 13 and Table 14. If the F value of a factor satisfies F > F_0.01_(3,3), this factor presents a highly significant effect on the test results, denoted by “***”; if F_0.05_(3,3) < F < F_0.01_(3,3), the factor has a relatively significant influence, denoted by “**”; if F_0.1_(3,3) < F < F_0.05_(3,3), the factor shows a marginally significant influence, denoted by “*”; if F < F_0.1_(3,3), the influence of the corresponding factor is non-significant.

As presented in Table 8, the sand-binder ratio, slag content and water-binder ratio are the dominant factors affecting the fluidity of metakaolin-slag geopolymer mortar, followed by water glass modulus, while alkali equivalent exerts an insignificant influence on fluidity. According to Table 9 and Table 10, alkali equivalent and sand-binder ratio have significant effects on the 7-day flexural strength of mortar; the 7-day compressive strength is remarkably affected by alkali equivalent and slag content, and other factors show negligible impacts. As illustrated in Table 11 and Table 12, the 28-day flexural strength is only significantly influenced by the sand-binder ratio. For the 28-day compressive strength, alkali equivalent poses the most prominent effect, and slag content also delivers a significant influence. It can be concluded from Table 13 and Table 14 that slag content and alkali equivalent significantly affect the compressive-flexural strength ratio and drying shrinkage rate of mortar, acting as the critical parameters for regulating the toughness and volume stability of mortar.

### 3.3. Comprehensive Analysis and Optimization of the Mix Proportion

#### 3.3.1. Optimal Mix Proportion Design

Range and average effect analysis often use a single control indicator only, such as compressive strength or fluidity for mix proportion design; therefore, the mortar’s mix proportion obtained by this method may result in poor comprehensive performance. In addition, to obtain the mortar mix proportion with the best comprehensive performance, the matrix analysis method is employed for optimal mix proportion determination. The matrix analysis [51] is an objective data processing method. The test results from the direct observation analysis are processed by setting up a weight matrix for each indicator. In the analysis and processing, the test results are not used directly; instead, comprehensive calculations and comparisons are performed, primarily based on data from direct observational analyses.

Four additional key influence indicators were selected: compressive strength to characterize mechanical properties, fluidity to assess workability, compressive-to-flexural strength ratio to characterize brittleness, and drying shrinkage rate to characterize crack resistance. A multi-indicator analysis was performed to obtain a mortar mix proportion with excellent comprehensive performance. Based on the orthogonal test data, the weight matrices of the above four indicators were constructed, and the average was taken as the basis for comprehensive performance evaluation.

A three-layer structure matrix model was established based on the test results. The first layer is the indicator layer matrix M, the second layer corresponds to the factor layer matrix T, and the third layer represents the level layer matrix S. The calculation process is as follows in Equation (3):

(3) $$M=\left[\begin{array}{cccc} K_{11} & 0 & \ldots & 0\\ K_{12} & 0 & \ldots & 0\\ \ldots & \ldots & \ldots & \ldots \\ K_{1j} & 0 & \ldots & 0\\ 0 & K_{21} & \ldots & 0\\ 0 & K_{22} & \ldots & 0\\ \ldots & \ldots & \ldots & \ldots \\ 0 & K_{2j} & \ldots & 0\\ 0 & 0 & \ldots & K_{i1}\\ 0 & 0 & \ldots & K_{i2}\\ \ldots & \ldots & \ldots & \ldots \\ 0 & 0 & \ldots & K_{\mathrm{ij}} \end{array}\right]T=\left[\begin{array}{cccc} t_{1} & 0 & \ldots & 0\\ 0 & t_{2} & \ldots & 0\\ \ldots & \ldots & \ldots & \ldots \\ 0 & 0 & \ldots & t_{i} \end{array}\right]S=\left[\begin{array}{c} S_{1}\\ S_{2}\\ \ldots \\ S_{i} \end{array}\right]$$

In the formula, $k_{ij}$ is the average value of the test indicator at the j-th level of the factor $A_{i}$. If the indicator is larger, then $K_{ij}=k_{ij}$; if the indicator is, then $K_{ij}=1/k_{ij}$. $t_{i}=1/\sum_{j=1}^{n}K_{ij}$. Let $s_{i}$ be the range of factors $A_{i}$, then $S_{i}=s_{i}/\sum_{i=1}^{m}s_{i}$.

Based on the test data in Table 3 and Table 5, taking the 28 d compressive strength as an example, the three-layer matrix calculation process is as follows:

$$M_{1}=\left[\begin{array}{ccccc} 45.3 & 0 & 0 & 0 & 0\\ 55.1 & 0 & 0 & 0 & 0\\ 68 & 0 & 0 & 0 & 0\\ 74.2 & 0 & 0 & 0 & 0\\ 0 & 55.5 & 0 & 0 & 0\\ 0 & 57.4 & 0 & 0 & 0\\ 0 & 64.4 & 0 & 0 & 0\\ 0 & 65.2 & 0 & 0 & 0\\ 0 & 0 & 39.9 & 0 & 0\\ 00 & 0 & 50.5 & 0 & 0\\ 0 & 0 & 67.9 & 0 & 0\\ 0 & 0 & 75.3 & 0 & 0\\ 0 & 0 & 0 & 62.3 & 0\\ 0 & 0 & 0 & 61.9 & 0\\ 0 & 0 & 0 & 61.8 & 0\\ 0 & 0 & 0 & 56.6 & 0\\ 0 & 0 & 0 & 0 & 55.3\\ 0 & 0 & 0 & 0 & 59.7\\ 0 & 0 & 0 & 0 & 59.8\\ 0 & 0 & 0 & 0 & 67.7 \end{array}\right]\;T_{1}=\left[\begin{array}{ccccc} \frac{1}{242.5} & 0 & 0 & 0 & 0\\ 0 & \frac{1}{242.5} & 0 & 0 & 0\\ 0 & 0 & \frac{1}{242.5} & 0 & 0\\ 0 & 0 & 0 & \frac{1}{242.5} & 0\\ 0 & 0 & 0 & 0 & \frac{1}{242.5} \end{array}\right]\;S_{1}=\left[\begin{array}{c} 28.9/92.1\\ 9.7/92.1\\ 35.4/92.1\\ 5.7/92.1\\ 12.4/92.1 \end{array}\right]$$

Then, according to the three-layer matrix model, the weight matrix ω of the corresponding indicator is calculated, as shown in Equation (4). Finally, the average value of the weight matrices of all indicators is obtained, as shown in Equation (5). Table 15 shows the calculated data.

(4) ω=MTS

(5) $$\omega =\frac{\omega_{1}+\omega_{2}+\omega_{3}+\omega_{4}}{4}$$

In the equation, ω refers to the aggregated value of the four performance indicators; ω1 represents the weight matrix for the 28 d compressive strength; ω2 corresponds to the weight matrix for the 28 d compressive-to-flexural strength ratio; ω3 denotes the weight matrix for fluidity; and ω4 represents the weight matrix for the 28 d drying shrinkage rate.

Based on the above calculations, the level with the largest weight among the four factors is A1B4C4D4E2, i.e., the mix proportion of metakaolin-slag geopolymer mortar with relatively optimal comprehensive performance is as follows: slag content 20%, sodium silicate modulus 1.6, alkali equivalent 12%, water-to-binder ratio 0.49, sand-to-binder ratio 2. A verification supplementary experiment was carried out for this mix proportion, and the measured performance indicators were as follows: 28 d compressive strength 53.2 MPa, flexural strength 8.6 MPa, compressive-to-flexural strength ratio 6.2, interfacial flexural bond strength 6.3 MPa, drying shrinkage rate 0.075%, fluidity 215 mm. All indicators significantly exceed rigid repair mortar’s specification requirements (28 d compressive strength > 30 MPa, flexural strength > 6 MPa, compressive-to-flexural strength ratio < 7, drying shrinkage rate < 0.1%, and interfacial bond strength > 2 MPa).

#### 3.3.2. Multiple Linear Regression Analysis

Based on the orthogonal test results, multiple linear regression analysis of each mortar indicator was performed using the SPSS software (Version 27.0, IBM Corp., Armonk, NY, USA, 2020). Let Y represent the dependent variable, and let x1, x2, ……, xi represent the i independent variables influencing Y, respectively. The multiple linear regression equation for the orthogonal design factors is established as Equation (6):

(6) $$\begin{array}{l} Y_{1}=16.231+49.812x_{1}+18.087x_{2}+571.875x_{3}-86.375x_{4}+7.425x_{5}\\ Y_{2}=0.383+2.1x_{1}+3.925x_{2}+42.5x_{3}-15x_{4}+2.19x_{5}\\ Y_{3}=-241.913+92.375x_{1}+49.875x_{2}+81.25x_{3}+878.75x_{4}-43.15x_{5}\\ Y_{4}=-0.089+0.066x_{1}+0.036x_{2}-0.583x_{3}+0.305x_{4}+0.007x_{5} \end{array}$$

In the formula, Y_1_ is the 28 d compressive strength; Y_2_ is the 28 d flexural strength; Y_3_ is the fluidity; Y_4_ is the 28 d drying shrinkage rate; x_1_ is the slag content; x_2_ is the sodium silicate modulus; x_3_ is the alkali equivalent; x_4_ is the water-to-binder ratio; and x_5_ is the sand-to-binder ratio.

A correlation test was conducted on this regression equation. For the R test, the statistic R value is in the range of 0 < |R| ≤ 1. The goodness of fit of the multiple linear regression equation is evaluated by R^2^ (coefficient of determination) and adjusted R^2^. A model with R^2^ > 0.9 and a difference from the adjusted R^2^ of less than 0.1 has a good fit. For the F test, the *p*-value is required to be < 0.001. Overall, except for the 28 d flexural strength indicator, the multiple linear regression fit for the other three parameters is relatively high (Table 16).

### 3.4. Microscopic Analysis

To reveal the intrinsic mechanism behind the differences in the macroscopic mechanical properties of the metakaolin-slag geopolymer mortar at the micro level, representative specimens were selected based on the orthogonal test results in Table 3 to conduct microstructural research. Considering that alkali equivalent and slag content are the two most significant factors affecting the mortar’s various properties, this study selected four groups of specimens, X1, X4, X11, and X14, for microstructural analysis. Group X1 represents low slag content and low alkali equivalent conditions; Group X4 represents low slag content and high alkali equivalent conditions; Group X11 represents high slag content and low alkali equivalent conditions; and Group X14 represents high slag content and high alkali equivalent conditions. XRD and SEM-EDS were used to elucidate the underlying causes of the differences in the macroscopic performance from reaction mechanisms.

#### 3.4.1. Phase Analysis

**Figure 10:**
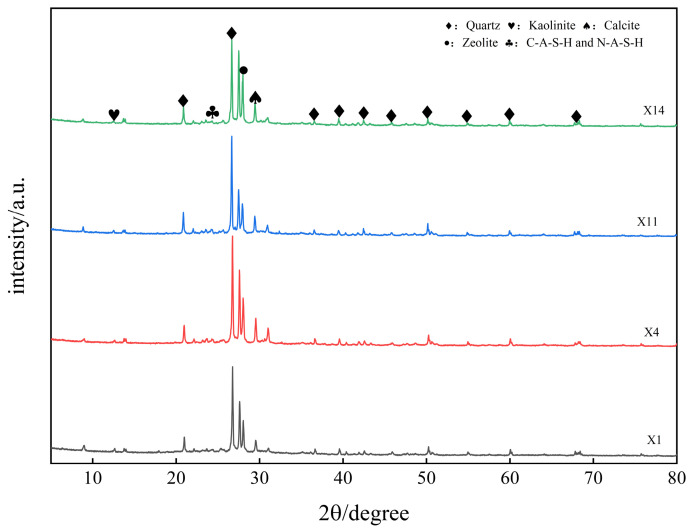
XRD patterns of different mix proportions at 28 d.

Figure 10 shows the XRD patterns of the mortar specimens with different mix proportions at 28 days of age (Groups X1, X4, X11, and X14). It can be seen from the figure that the XRD patterns of mortars with different mix proportions show similar diffraction peak shapes and positions, indicating that the product composition is consistent, and that the adjustment of mix proportions did not induce the formation of new hydration products. The quartz phase (SiO_2_) has the highest diffraction intensity, indicating that it has the largest content. This is attributed to the fact that natural river sand is used as a fine aggregate, and its main component is quartz. The aggregate has very low activity, does not participate in the hydration reaction, and remains stable in the system in its original crystalline form. The characteristic diffraction peak of calcite (CaCO_3_) appears near 2θ = 29°. This can be attributed to two primary reasons: first, during the hardening process of the mortar, Ca^2+^ that did not participate in the hydration reaction undergoes carbonation with CO_2_ in the air to form CaCO_3_ [52]. Second, a small amount of limestone debris may be mixed in during the mining and sieving of the material [53], resulting in calcite existing as an impurity. Calcite has no reactivity and does not participate in the geopolymer reaction. In addition, a weak characteristic diffraction peak of kaolinite was observed near 2θ = 12.5°. Kaolinite is a common residual phase in metakaolin, and its characteristic peak intensity can indirectly reflect the degree of reaction of metakaolin [54]. Comparing the intensity of the characteristic peak of kaolinite near 12.4° among the four groups, the characteristic peak of Group X1 is stronger than those of the other three groups, and this group has the lowest values of slag content and alkali equivalent set in the test, followed by Group X11, which has the lowest alkali equivalent setting. This phenomenon indicates that increasing both the slag content and the alkali equivalent can effectively promote metakaolin dissolution reaction and the geopolymer process, thereby reducing the residual amount of kaolinite in the system.

The broad and diffuse diffraction peak appearing in the 2θ = 20°–35° range originates from the amorphous or low-crystallinity characteristics of the geopolymer gel. The internal silicoaluminate tetrahedra are disordered and crosslinked. The very low crystallinity causes the diffraction signal to be dispersed without a clear characteristic peak position. The main components are C-(A)-S-H and N-A-S-H [55]. In addition, characteristic peaks of low-crystallinity zeolite phases (CaAl_2_Si_2_O_8_·4H_2_O, Na_2_Al_2_Si_3_O_10_·2H_2_O) can be observed near 2θ = 28.2°. Zeolites are mainly transformed from amorphous C-A-S-H and N-A-S-H gels. Their basic structural units are siloxane tetrahedra (SiO_4_^4−^) and aluminate tetrahedra (AlO_4_^5−^), which are alternately connected by sharing oxygen atoms to form a three-dimensional network skeleton [56,57]. Zeolite crystals can form a tight bond with the amorphous geopolymer gel, thereby reducing interface defects. Simultaneously, zeolite crystals can fill the pores of the gel network, optimize the microstructure’s compactness, and thus enhance the system’s strength and stability. The broad amorphous diffraction peaks and characteristic peak intensities of zeolite phases in Groups X4 and X14 are significant, which is in good agreement with the compressive and flexural strength test results of the two groups of specimens in Table 3.

#### 3.4.2. Microstructural Analysis

**Figure 11:**
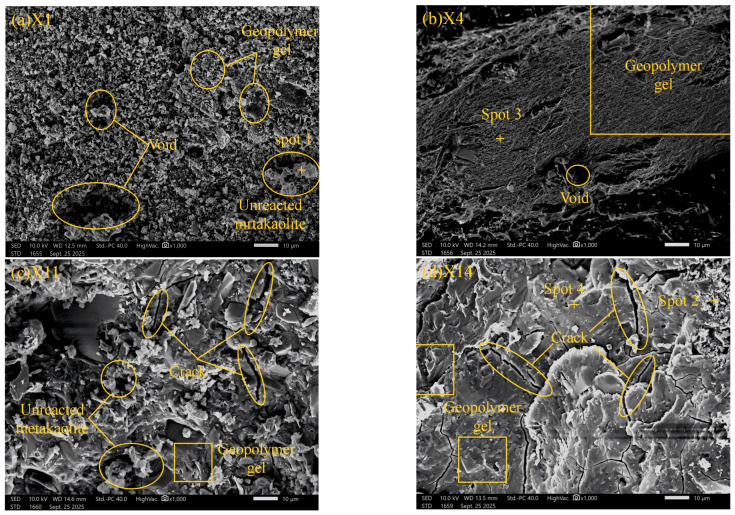
SEM micrographs of different mix proportions at 28 d.

SEM images of the mortar specimens at 28 d age are shown in Figure 11, with a magnification of 1000×. The figure shows that the main hydration products of the metakaolin-slag geopolymer mortar are flocculent and blocky amorphous gels. Comparing the images of the four groups in Figure 9, a large amount of unreacted metakaolin particles still remains in Groups X1 and X11, resulting in relatively low mechanical strength for these two groups. The alkali equivalent of these two groups was set at the lowest value of 6%, whereas Groups X4 and X14 used higher alkali equivalents, and no obvious unreacted metakaolin particles were observed in their images. This phenomenon confirms that increasing the alkali equivalent can promote the dissolution reaction of metakaolin and the smooth progress of the geopolymer reaction. Because the alkalinity of Groups X1 and X11 was insufficient to drive the full reaction of metakaolin and slag, relatively few cementitious products were generated in the system. In the image of Group X1, the number of cementitious products was very small, and there were many voids and pores in the system, with a 28 d compressive strength of only 15.8 MPa.

Further comparing the test results and microstructural characteristics of Groups X1, X11, and X14, it can be seen that a denser microstructure is formed inside the specimen with the increase in slag content, indicating that the geopolymer reaction in the system becomes more complete. The alkali activation activity of slag is significantly higher than that of metakaolin. The action of the alkaline activator can quickly release the active components of silicon and aluminum, which not only promotes the dissolution reaction of metakaolin particles but also accelerates the geopolymer reaction process. In addition, the CaO contained in slag can release a large amount of Ca^2+^ in an alkaline environment, and Ca^2+^ is a core component for forming high-strength C-A-S-H gel. This gel interweaves with the N-A-S-H gel generated by metakaolin reaction to form a composite interpenetrating network structure. Compared with a single gel, this composite gel system has higher mechanical strength and structural stability, as well as better pore-filling ability, thus making the specimen’s microstructure [58]. Therefore, Group X14 specimens showed the highest compressive strength, reaching 86.9 MPa. However, v, and the Ca^2+^ provided by slag accumulates excessively as the content increases, which increases the calcium-silicon ratio (Ca/Si) of the C-(A)-S-H gel and increases the structural brittleness [59]. Simultaneously, it inhibits the flexible regulation effect of N-A-S-H gel, reduces the crack resistance toughness of the composite gel network, and causes more cracks and micro-defects in the internal structure, making the specimen prone to water loss, as shown in Figure 11d. This not only significantly increases the shrinkage rate of the specimen but also decreases the flexural strength, even though the amount of gel products increases. Although the specimens of Group X14 had the best compressive strength, reaching 86.9 MPa at 28 d, their 28 d flexural strength was only 9.6 MPa, which was lower than that of Group X4 (11 MPa).

**Figure 12:**
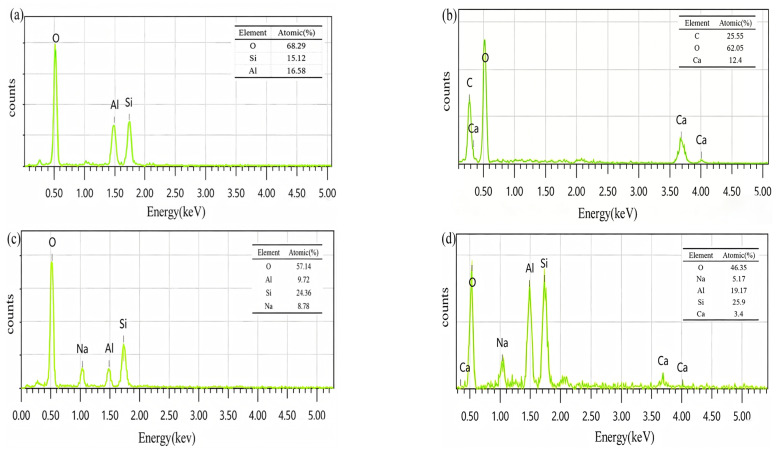
EDS spectra of different products (Spot 1, Spot 2, Spot 3, and Spot 4 in (**a**–**d**), respectively).

EDS was used to analyze its elements, and the energy-dispersive spectroscopy point scan results of the mortar are shown in Figure 12. To ensure the representativeness of EDS analysis, the selected test points covered typical regions with distinct morphologies in the matrix. Comparative analysis indicates that the elemental distribution of reaction products in the system mainly presents four typical patterns. Energy spectrum point 1 mainly detected three elements: Al, Si, and O, with an Al/Si atomic ratio of approximately 1.0. This area corresponds to unreacted metakaolin particles or their reaction residues. The surface of some metakaolin particles does not participate in the reaction as an amorphous aluminosilicate source, and the interior still retains the original compositional characteristics. Energy spectrum point 2 mainly detected three elements: Ca, C, and O, which may be related to slight carbonation during specimen preparation or testing, resulting in a small amount of calcium carbonate distributed on the matrix surface [52].

Energy spectrum point 3 detected four elements: Na, Al, Si, and O, with a Si/Al atomic ratio of approximately 2.0, and a prominent Na element peak. This is a typical characteristic of the N-A-S-H gel. The main reaction product of the metakaolin-based geopolymer is N-A-S-H gel. In its three-dimensional network structure, Na^+^ acts to balance the negative charge of the framework. This gel is widely distributed and constitutes the main matrix skeleton of the matrix. Energy spectrum point 4 detected multiple elements, including Na, Ca, Al, Si, and O, showing the characteristics of N-(C-)A-S-H, i.e., a composite region where N-A-S-H and C-A-S-H gels coexist. This indicates that in the metakaolin-slag composite system, the two types of gels work together to form a denser microstructural network, which is the fundamental reason why the binary geopolymer achieves excellent mechanical properties. Comprehensive energy-dispersive X-ray diffraction (EDS) analysis results reveal that the gel products in the metakaolin-slag geopolymer system are not of a single type but are composed of N-A-S-H, C-A-S-H, and their composite gels, which are consistent with the products analyzed in the XRD patterns. The formation ratio and distribution characteristics of each type of gel are influenced by factors such as raw material composition, alkali activation conditions, and modifier content.

### 3.5. Sustainability Analysis

**Figure 13:**
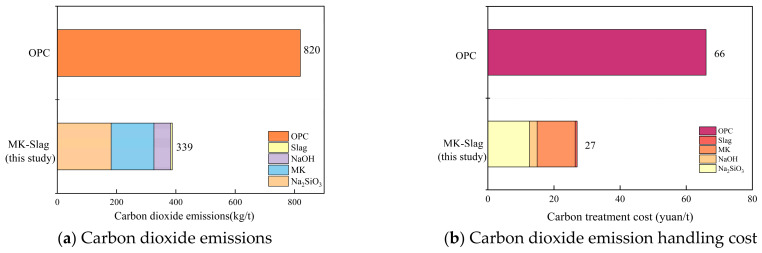
Comparison of CO_2_ emissions and treatment costs of MK-Slag (this) and OPC.

The sustainability of the geopolymer mortar was analyzed based on the optimal mix proportion determined in this study, combined with the carbon emission data of raw materials, carbon treatment prices, and relevant specifications [60]. Ordinary Portland cement mortar (OPC) with similar strength to the geopolymer mortar of this study was selected as a reference for comparison. In terms of carbon emissions, the geopolymer mortar shows a significant low-carbon advantage. According to calculations, the carbon emission of the geopolymer mortar prepared in this study is 339 kg/t, whereas the ordinary cement mortar is 820 kg/t (Figure 13). The difference in carbon emission intensity directly translates into the carbon cost side, calculated based on the average national carbon market allowance (CEA) price of CNY 80 per ton; the carbon emission cost per ton of cementitious materials is reduced by CNY 39. This advantage is mainly due to the fact that geopolymer mortar replaces cement clinker with industrial solid waste slag and highly reactive mineral metakaolin. Although the alkaline activator produces certain carbon emissions, its overall emissions are still far lower than the energy consumption of cement clinker calcination and grinding.

Although the geopolymer mortar has already shown good low-carbon characteristics, its carbon emissions still have room for further optimization. The main contributor to carbon emissions is sodium silicate, accounting for nearly 50% of the total. The sodium silicate production process has high energy consumption and is the main source of carbon emissions. The calcination process of metakaolin also contributes to a certain proportion of carbon emissions. Therefore, reducing the amounts of MK and Na_2_SiO_3_ while maintaining the desired material properties is a key way to further improve the material’s sustainability. Overall, the geopolymer mortar prepared in this study not only ensures excellent performance (The 28-day compressive strength is approximately 25% higher than that of PO 42.5 Ordinary Portland cement mortar, and its flexural strength is increased by roughly 20%. Moreover, the bonding strength reaches three times the limit specified by relevant specifications.) but also has significant environmental benefits, aligning with the development direction of green and low-carbon construction materials (Table 17).

(7) $$E_{total}=\sum_{i=1}^{n}\left(m_{i}\times EF_{i}\right){\;\;\;\;\;\;\;E}_{unit}=E_{total}/M_{total}$$

In the formula, E_total_: carbon emissions of the system, unit: kg CO_2_ e (carbon dioxide equivalent); m_i_: amount of the i-th raw material, unit: kg; EF_i_: carbon emission factor of the i-th raw material per unit, unit: kg CO_2_ e/kg; E_unit_: carbon emissions per unit mass of cementitious material, unit: kg CO_2_e/kg; M_total_: total mass of cementitious material in the system, unit: kg.

## 4. Conclusions

This study systematically investigated the influence of mix proportion parameters on geopolymer mortar performance. An orthogonal experimental design combined with microscopic characterization techniques was adopted for analysis. Metakaolin and slag were selected as silicoaluminous raw materials, and water glass was used as the alkali activator. In compliance with relevant specification requirements, key indicators including fluidity, mechanical strength, toughness and crack resistance were comprehensively considered, and the mixing ratio of mortar with the optimal comprehensive performance was finally determined. Based on the above experimental results and analysis, the main conclusions are drawn as follows:

(1) From the range R and mean square ratio F, there are differences in the order of influence on different indicators of metakaolin-slag geopolymer mortar. For compressive strength, alkali equivalent is the most significant factor (largest R and F), followed by slag content; sand-to-binder ratio and sodium silicate modulus show the smallest R and F values, indicating negligible influence. For flexural strength, sand-to-binder ratio and alkali equivalent are the dominant factors, jointly determining flexural performance. For the compressive-to-flexural strength ratio (toughness), alkali equivalent and slag content are the key controlling factors. For fluidity, water-to-binder ratio and sand-to-binder ratio exert the strongest effects, followed by slag content and then sodium silicate modulus. For drying shrinkage, slag content and alkali equivalent play decisive roles, which are crucial for mortar volume stability.

(2) From the perspective of the influence pattern of the average value k, both compressive strength and flexural strength increase significantly with an increase in the amount of alkali equivalent. The flexural strength first increases and then decreases with an increase in slag content, reaching a peak at 60–80% content. Fluidity increases with the increase in slag content, water-to-binder ratio, and sodium silicate modulus, and decreases with the increase in sand-to-binder ratio. The drying shrinkage rate increases with the increase in slag content, sodium silicate modulus, water-to-binder ratio, and sand-to-binder ratio, and decreases with the increase in the alkali equivalent.

(3) Based on the orthogonal test results, considering multiple indicators including compressive strength, compressive-to-flexural strength ratio, fluidity, and drying shrinkage rate, the optimal mix proportion was determined as follows: slag content 20%, sodium silicate modulus 1.6, alkali equivalent 12%, water-to-binder ratio 0.49, and sand-to-binder ratio 2:1. The geopolymer mortar prepared under this combination has a 28-day compressive strength of 53.2 MPa, flexural strength of 8.6 MPa, compressive-to-flexural ratio of 6.2, interfacial flexural bonding strength of 6.3 MPa, drying shrinkage of 0.075%, and workability of 215 mm, which can ensure both fluidity and meet the requirements of repair mortar, achieving the best overall performance.

(4) Microstructural test results show that the main cementitious products of the metakaolin-slag system are N-A-S-H gel and C-(A)-S-H gel. Increasing both slag content and alkali equivalent can effectively promote the dissolution of aluminosilicate raw materials, thereby enhancing the degree of geopolymer reaction. However, when the slag content is too high, although it can still increase the amount of geopolymer gel formed, it will simultaneously cause more and larger micro-cracks inside the mortar, ultimately destroying the integrity of the system’s internal structure.

(5) The MK-Slag geopolymer mortar prepared in this study has higher sustainability than traditional cement mortar of similar strength, and its carbon emissions and carbon treatment costs can be reduced by about 60%.

## Figures and Tables

**Table 1:** The main chemical composition and content of metakaolin and slag.

Content	SiO_2_	Al_2_O_3_	Fe_2_O_3_	CaO	K_2_O	MgO	Na_2_O	Other
MK (%)	53.70	43.60	0.89	0.08	0.43	0.05	0.05	1.2
Slag (%)	33.06	15.04	1.29	39.29	0.56	9.66	0.56	0.54

**Table 2:** Sieve analysis test results of medium-sized sand.

Size (mm)	4.75	2.36	1.18	0.6	0.3	0.15
Cumulative surplus (%)	3.7	12.9	26.5	48	81.8	93.5
Technical requirements (%)	0–10	0–25	10~50	41–70	70~92	90–100

**Table 3:** The orthogonal experimental design.

Level	A Slag Content (%)	B Water Glass Modulus	C Alkali Equivalent (%)	D Water-Binder Ratio	E Sand-Binder Ratio
1	20	1.0	6	0.43	1.5
2	40	1.2	8	0.45	2
3	60	1.4	10	0.47	2.5
4	80	1.6	12	0.49	3

**Table 4:** Orthogonal test data results.

Group	Factor	Fluidity (mm)	Flexural Strength (MPa)		Compressive Strength (MPa)		28 d Compressive to Flexural Strength Ratio	28 d Shrinkage Rate (%)
			7 d	28 d	7 d	28 d		
X1	A1B1C1D1E1	132	2.4	3.6	11.2	15.8	4.4	0.060
X2	A1B2C2D2E2	158	6.3	6.8	32.8	41.3	6.1	0.065
X3	A1B3C3D3E3	165	7.5	8.9	48.0	56.6	6.4	0.071
X4	A1B4C4D4E4	155	10.3	11.0	61.9	67.5	6.1	0.075
X5	A2B1C2D3E4	143	9.5	9.7	52.6	57.0	5.9	0.106
X6	A2B2C1D4E3	184	4.0	4.6	23.7	26.2	5.7	0.120
X7	A2B3C4D1E2	170	8.9	10.3	72.3	74.2	7.2	0.075
X8	A2B4C3D2E1	220	7.3	8.2	56.2	62.8	7.7	0.095
X9	A3B1C3D4E2	221	7.7	7.8	55.7	65.1	8.3	0.090
X10	A3B2C4D3E1	225	7.9	7.5	69.0	75.2	10.0	0.075
X11	A3B3C1D2E4	150	7.6	10.4	59.5	59.3	5.7	0.125
X12	A3B4C2D1E3	176	8.5	10.7	71.5	72.3	6.8	0.111
X13	A4B1C4D2E3	183	9.4	9.6	75.7	84.1	8.8	0.088
X14	A4B2C3D1E4	140	9.0	9.6	73.2	86.9	9.1	0.090
X15	A4B3C2D4E1	270	6.6	6.9	51.6	67.5	9.8	0.125
X16	A4B4C1D3E2	245	6.5	8.6	57.6	58.3	6.8	0.143

**Table 5:** Range analysis of fluidity.

Factor	A: Slag Content	B: Water Glass Modulus	C: Alkali Equivalent	D: Water-to- Binder Ratio	E: Sand-to- Binder Ratio
k1	152.5	169.8	177.8	154.5	211.8
k2	179.3	176.8	186.8	177.8	198.5
k3	193.0	188.8	186.5	194.5	177.0
k4	209.5	199.0	183.3	207.5	147.0
R	57.0	29.0	9.0	53.0	64.8

**Table 6:** Range analysis of the mechanical strength.

Item	Factor	k1	k2	k3	k4	R	Sequence
7 d Flexural strength	A	6.6	7.4	7.9	7.8	1.3	C > E > B > A > D
	B	7.3	6.8	7.7	8.2	1.4	
	C	5.1	7.7	7.9	9.1	4	
	D	7.2	7.7	7.9	7.2	0.7	
	E	6.0	7.3	7.5	9.1	3.1	
7 d Compressive strength	A	38.5	51.2	63.9	64.5	26	C > A > E > B > D
	B	48.8	49.7	57.9	61.8	13	
	C	38.0	52.1	58.3	69.7	31.7	
	D	57.0	56.0	56.8	48.2	8.8	
	E	47.0	54.6	54.7	61.8	14.8	
28 d Flexural strength	A	7.6	8.2	9.1	8.7	1.5	E > C > B > A > D
	B	7.7	7.1	9.1	9.6	2.5	
	C	6.8	8.5	8.6	9.6	2.8	
	D	8.6	8.8	8.7	7.6	1.2	
	E	6.6	8.4	8.5	10.2	3.6	
28 d Compressive strength	A	45.3	55.1	68.0	74.2	28.9	C > A > E > B > D
	B	55.5	57.4	64.4	65.2	9.7	
	C	39.9	59.5	67.9	75.3	35.4	
	D	62.3	61.9	61.8	56.6	5.7	
	E	55.3	59.7	59.8	67.7	12.4	
28 d Compressive to flexural strength ratio	A	5.8	6.6	7.7	8.5	2.9	A > C > E > B > D
	B	6.9	7.7	7.3	6.9	0.9	
	C	5.7	7.2	7.9	8.0	2.4	
	D	6.9	7.1	7.3	7.5	0.6	
	E	8	7.1	6.9	6.7	1.3	

**Table 7:** Range analysis of drying shrinkage rate.

Factor	A: Slag Content	B: Water Glass Modulus	C: Alkali Equivalent	D: Water-to-Binder Ratio	E: Sand-to-Binder Ratio
k1	0.0678	0.0860	0.1120	0.084	0.0888
k2	0.0990	0.0875	0.1018	0.0933	0.0933
k3	0.1003	0.0990	0.0865	0.0988	0.0975
k4	0.1115	0.1060	0.0783	0.1025	0.099
R	0.0438	0.0200	0.0338	0.0185	0.0103

**Table 8:** Analysis of variance for fluidity.

Influence Factor	Variance Analysis					
	Dev-Sq	DOF	F-Ratio	F-Critical Value	*p*-Value	Significance
A	6981.19	3	33.13	F_0.1_(3,3) = 5.39	<0.01	***
B	2009.69	3	9.54	F_0.05_(3,3) = 9.28	<0.05	**
C	210.69	3		F_0.01_(3,3) = 29.46	>0.1	
D	6284.19	3	29.83		<0.01	***
E	9590.19	3	45.52		<0.01	***
Error	210.69	3				

Note: If F-ratio is bigger than F_0.01_ = 29.46, the significance is ***; if the F-ratio is bigger than F_0.05_ = 9.28, the significance is **.

**Table 9:** Analysis of variance for 7 d flexural strength.

Influence Factor	Variance Analysis					
	Dev-Sq	DOF	F-Ratio	F-Critical Value	*p*-Value	Significance
A	4.35	3	3.09	F_0.1_(3,3) = 5.39	<0.1	
B	3.97	3	2.82	F_0.05_(3,3) = 9.28	>0.1	
C	33.87	3	24.06	F_0.01_(3,3) = 29.46	<0.05	**
D	1.41	3			>0.1	
E	17.18	3	12.21		<0.05	**
Error	1.41	3				

Note: If the F-ratio is bigger than F_0.05_ = 9.28, the significance is **.

**Table 10:** Analysis of variance for 7 d compressive strength.

Influence Factor	Variance Analysis					
	Dev-Sq	DOF	F-Ratio	F-Critical Value	*p*-Value	Significance
A	1828.45	3	8.57	F_0.1_(3,3) = 5.39	<0.1	*
B	480.35	3	2.25	F_0.05_(3,3) = 9.28	>0.1	
C	2096.28	3	9.83	F_0.01_(3,3) = 29.46	<0.05	**
D	213.32	3			>0.1	
E	413.91	3	1.94		>0.1	
Error	213.32	3				

Note: If the F-ratio is bigger than F_0.05_ = 9.28, the significance is **; if F-ratio is bigger than F _0.1_ = 5.39, the significance is *.

**Table 11:** Analysis of variance for 28 d flexural strength.

Influence Factor	Variance Analysis					
	Dev-Sq	DOF	F-Ratio	F-Critical Value	*p*-Value	Significance
A	5.14	3	1.43	F_0.1_(3,3) = 5.39	>0.1	
B	16.71	3	4.64	F_0.05_(3,3) = 9.28	>0.1	
C	16.26	3	4.52	F_0.01_(3,3) = 29.46	>0.1	
D	3.6	3			>0.1	
E	26.3	3	7.31		<0.1	*
Error	3.6	3				

Note: If F-ratio is bigger than F _0.1_ = 5.39, the significance is *.

**Table 12:** Analysis of variance for 28 d compressive strength.

Influence Factor	Variance Analysis					
	Dev-Sq	DOF	F-Ratio	F-critical Value	*p*-Value	Significance
A	2016.96	3	22.82	F_0.1_(3,3) = 5.39	<0.05	**
B	288.31	3	3.26	F_0.05_(3,3) = 9.28	>0.1	
C	2787.31	3	31.54	F_0.01_(3,3) = 29.46	>0.1	***
D	88.37	3			>0.1	
E	317.13	3	3.59		>0.1	
Error	88.37	3				

Note: If F-ratio is bigger than F_0.01_ = 29.46, the significance is ***; if the F-ratio is bigger than F_0.05_ = 9.28, the significance is **.

**Table 13:** Analysis of variance for 28 d compressive-to-flexural strength ratio.

Influence Factor	Variance Analysis					
	Dev-Sq	DOF	F-Ratio	F-Critical Value	*p*-Value	Significance
A	18.85	3	23.55	F_0.1_(3,3) = 5.39	<0.05	**
B	2.1	3	2.63	F_0.05_(3,3) = 9.28	>0.1	
C	14.16	3	17.7	F_0.01_(3,3) = 29.46	<0.05	**
D	0.8	3			>0.1	
E	3.74	3	4.69		>0.1	
Error	0.8	3				

Note: If the F-ratio is bigger than F_0.05_ = 9.28, the significance is **.

**Table 14:** Analysis of variance for 28 d drying shrinkage rate.

Influence Factor	Variance Analysis					
	Dev-Sq	DOF	F-Ratio	F-Critical Value	*p*-Value	Significance
A	0.00423	3	16.58	F_0.1_(3,3) = 5.39	<0.05	**
B	0.00109	3	4.29	F_0.05_(3,3) = 9.28	>0.1	
C	0.00275	3	10.76	F_0.01_(3,3) = 29.46	<0.05	**
D	0.00078	3	3.04		>0.1	
E	0.00026	3			<0.1	
Error	0.00026	3				

Note: If the F-ratio is bigger than F_0.05_ = 9.28, the significance is **.

**Table 15:** Comprehensive analysis of the orthogonal test.

Factor	ω_1_	ω_2_	ω_3_	ω_4_	ω	Optimal Levels
A1	0.0586	0.1083	0.0557	0.1172	0.0831	A1
A2	0.0713	0.0952	0.0652	0.0805	0.0816	
A3	0.0880	0.0816	0.0703	0.0797	0.0803	
A4	0.0960	0.0730	0.0765	0.0711	0.0795	
B1	0.0241	0.0289	0.0315	0.0434	0.0284	B4
B2	0.0249	0.0259	0.0328	0.0424	0.0273	
B3	0.0279	0.0273	0.0350	0.0377	0.0293	
B4	0.0283	0.0289	0.0369	0.0352	0.0308	
C1	0.0632	0.0919	0.0102	0.0554	0.0648	C4
C2	0.0801	0.0728	0.0107	0.0608	0.0592	
C3	0.1076	0.0663	0.0107	0.0713	0.0628	
C4	0.1193	0.0654	0.0105	0.0795	0.0652	
D1	0.0159	0.0193	0.0525	0.0410	0.0268	D4
D2	0.0158	0.0188	0.0603	0.0369	0.0284	
D3	0.0158	0.0182	0.0660	0.0348	0.0296	
D4	0.0144	0.0178	0.0701	0.0334	0.0300	
E1	0.0307	0.0358	0.0880	0.0216	0.0476	E2
E2	0.0331	0.0404	0.0826	0.0207	0.0491	
E3	0.0331	0.0415	0.0735	0.0196	0.0471	
E4	0.0376	0.0428	0.0610	0.0194	0.0461	

**Table 16:** Random F-test results of multiple linear regression equation parameters for each parameter in the orthogonal test.

Regression Equation Parameters	R Test				F Test	
	R	R^2^	Adjusted R^2^	Difference	F	*p*
Y1	0.972	0.945	0.918	0.033	34.696	<0.001
Y2	0.908	0.825	0.737	0.088	9.398	0.002
Y3	0.986	0.971	0.957	0.014	67.716	<0.001
Y4	0.951	0.905	0.857	0.048	19.015	<0.001

**Table 17:** Life cycle data of raw materials.

Material	Carbon Dioxide Emissions (kg/t)	Unit CEA Processing Cost (Yuan/t)	Explanation
OPC	820	66	General Benchmark Values for the Cement Industry
MK	280	22.4	Mining, low-temperature calcination, grinding
Slag	60	4.8	Energy consumption of powder processing
Na_2_SiO_3_	480	38.4	Covers the entire process of soda ash production and preparation
NaOH	1120	89.6	Average value of the industrial caustic soda industry

## Data Availability

The original contributions presented in this study are included in the article. Further inquiries can be directed to the corresponding author.
